# Selective protection of normal cells from chemotherapy, while killing drug-resistant cancer cells

**DOI:** 10.18632/oncotarget.28382

**Published:** 2023-03-11

**Authors:** Mikhail V. Blagosklonny

**Affiliations:** ^1^Roswell Park Comprehensive Cancer Center, Buffalo, NY 14263, USA

**Keywords:** oncology, resistance, cyclotherapy, trilaciclib, rapamycin

## Abstract

Cancer therapy is limited by toxicity in normal cells and drug-resistance in cancer cells. Paradoxically, cancer resistance to certain therapies can be exploited for protection of normal cells, simultaneously enabling the selective killing of resistant cancer cells by using antagonistic drug combinations, which include cytotoxic and protective drugs. Depending on the mechanisms of drug-resistance in cancer cells, the protection of normal cells can be achieved with inhibitors of CDK4/6, caspases, Mdm2, mTOR, and mitogenic kinases. When normal cells are protected, the selectivity and potency of multi-drug combinations can be further enhanced by adding synergistic drugs, in theory, eliminating the deadliest cancer clones with minimal side effects. I also discuss how the recent success of Trilaciclib may foster similar approaches into clinical practice, how to mitigate systemic side effects of chemotherapy in patients with brain tumors and how to ensure that protective drugs would only protect normal cells (not cancer cells) in a particular patient.

## INTRODUCTION

No cancer cell, no matter how resistant it is, can survive chemotherapy in a cell culture. In the organism, however, therapy of cancer is limited by killing or damaging normal cells. Selective protection of normal cells from chemotherapy would increase the therapeutic window, improving the therapeutic outcome. Needless to say, reduction of side effects and better quality of life are very important for a cancer patient.

The challenge is to ensure that protection of normal cells is selective or, in other words, that cancer cells are not protected. Here, we will discuss approaches to ensure selectivity. We will also discuss how multi-drug combinations could be designed to be antagonistic in normal cells and synergistic in cancer cells. Pre-clinical studies in paired cell lines and mice demonstrated that normal cells can be selectively protected. However, until recently, there was little hope of clinical application because this research was little known and seemed impracticable and obscure. The situation is radically changing now because, based in part on the outstanding work of Norman Sharpless and co-workers [1, 2], the CDK4/6 inhibitor Trilaciclib was successfully introduced for myeloprotection against chemotherapy in lung cancer. This success creates the opportunity for clinical translation of the entire concept: selective protection of normal cells by exploiting drug resistance of cancer cells.

To start with, some cancers are intrinsically resistant to anticancer drugs [3, 4]. Furthermore, apoptosis avoidance, mitogenic self-sufficiency and insensitivity to anti-proliferative stimuli are hallmarks of cancer [5]. Killing sensitive cancer cells, an initially effective cancer therapy, inevitably selects for acquired resistance. Resistant clones tend to be aggressive due to acquiring additional oncogenic mutations. This is how therapy fails.

In 1999, it was suggested to exploit resistance instead of its reversal [6].

### Solving the puzzle

Can we kill drug-resistant cells while sparing sensitive cells?

**Figure 1:**
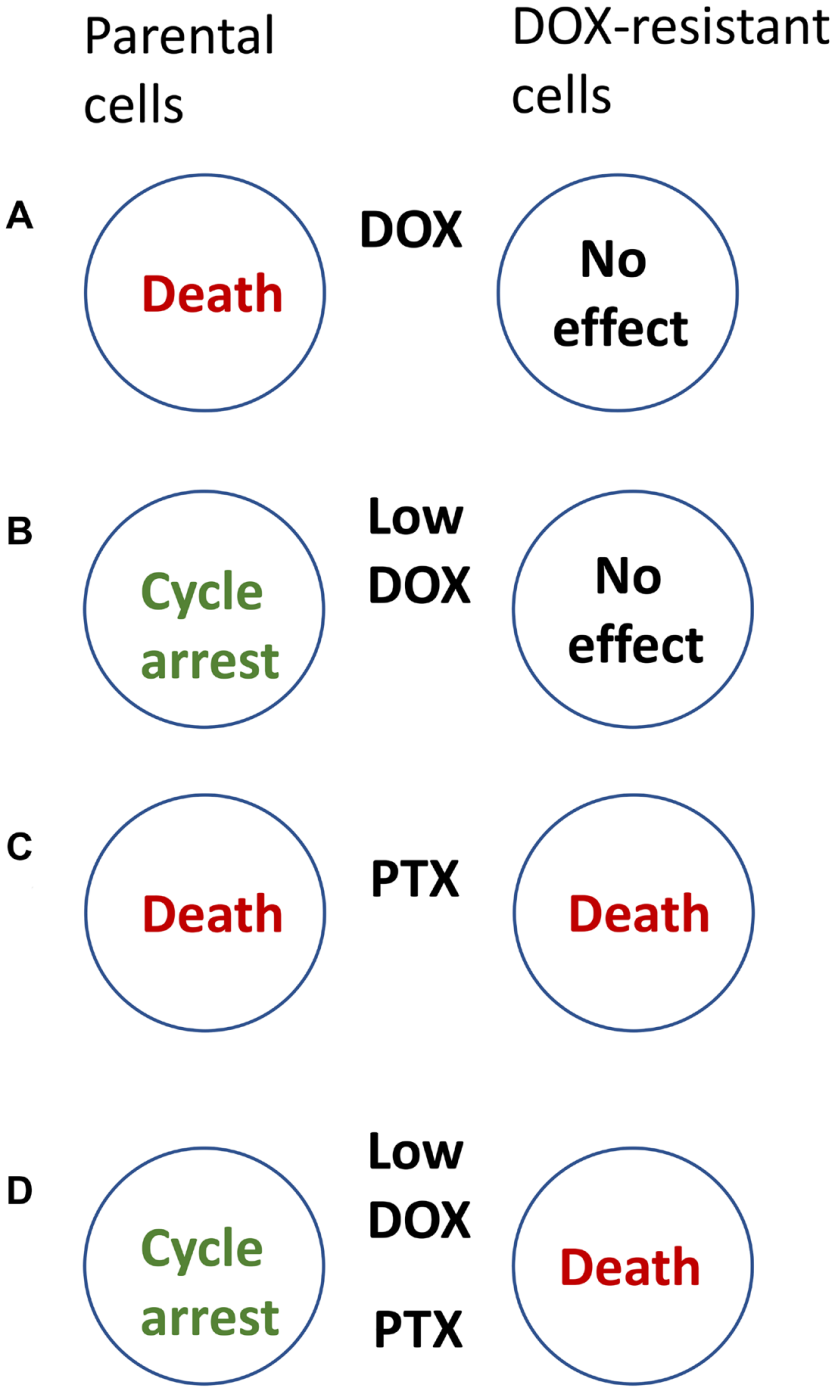
How to kill DOX-resistant cells, sparing sensitive cells. (**A**) Doxorubicin (DOX) kills parental sensitive cells, sparing resistant cells. (**B**) At low doses, DOX (Low-DOX) causes cell-cycle arrest in parental (sensitive) cells only. (**C**) Paclitaxel (PTX) kills both parental and DOX-resistant cells. (**D**) A combination of Low-Dox and PTX kills DOX-resistant cells, sparing parental (sensitive) cells.

Consider paired cell lines: the parental (sensitive) cancer cell line and drug-resistant cells selected for resistance to doxorubicin (DOX). (In a clinical analogy, the emergence of DOX-resistance cancer cells means therapeutic failure). DOX kills parental cells and spares DOX-resistant cells (Figure 1A). The task is, in contrast, to kill DOX-resistant cells and spare parental cells.

To avoid killing of parental cells, we may lower concentrations of DOX (Low-DOX), which then causes cell-cycle arrest in parental cells instead of cell death. However, Low-DOX cannot affect DOX-resistant (Figure 1B).

DOX-resistant cells, used in the study [6], were still sensitive to some other drugs, including Taxol (paclitaxel, PTX), which kills proliferating cells in mitosis. As expected, PTX kills both parental and resistance cells (Figure 1C). But the goal is to kill DOX-resistant cells only. At first glance, the task is impossible.

Selective killing of resistant cells cannot be achieved by one single drug. It is achievable by combining Low-DOX and PTX (Figure 1D). Low-DOX causes cell-cycle arrest in parental cells but not in Dox-resistant cells, which continue proliferation. Then, PTX kills these proliferating cells in mitosis, whereas parental cells are protected by low-DOX-induced cell-cycle arrest. Thus, a combination of low DOX and PTX kills DOX-resistant cells only [6].

Combination of low-Dox followed by PTX is semi-antagonistic: low-DOX prevents cell death by PTX. Secondly, the target cells are resistant to DOX and sensitive to PTX, whereas the protected cells are sensitive to both drugs [6].

### Selective killing Pgp/MRP-expressing cells

**Figure 2:**
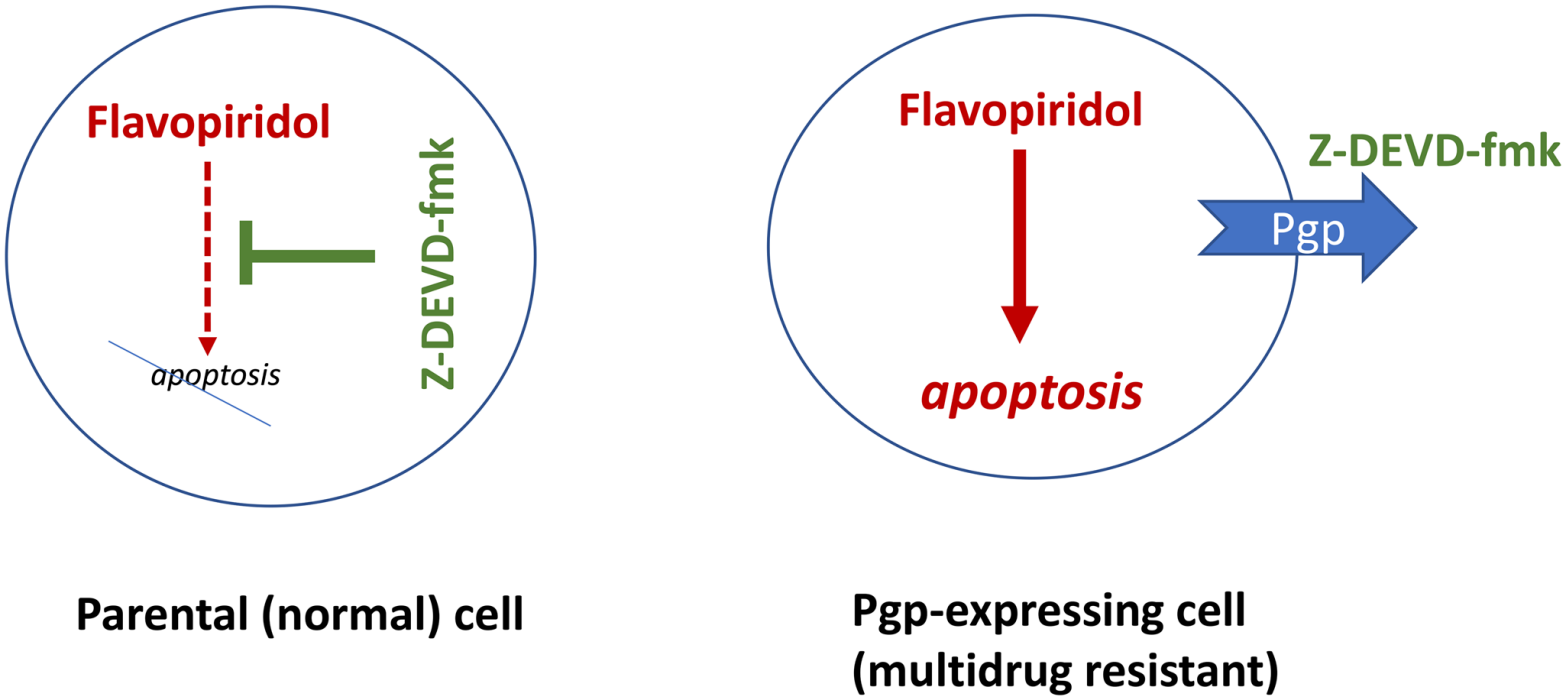
Caspase inhibitors (CI) selectively protect normal cells from chemotherapy-induced apoptosis, without protection multidrug-resistant cancer cells. Flavopiridol (shown in red, as a cytotoxic/lethal drug) can induce apoptosis. The caspase inhibitor Z-DEVD-fmk (green) can block apoptosis but multidrug-resistant cancer cells pump it out.

The simplest combination that selectively kills multidrug-resistant (MDR)-cells is an antagoistic combination including an (a) apoptosis-inducing drug and (b) inhibitor of apoptosis [7]. The apoptosis-inducing drug should NOT be a substrate of Pgp/MRP1. For example, flavopiridol (Alvocidib), a pan-CDK inhibitor, induces apoptosis in both parental and multidrug-resistant HL60 cells. The anti-apoptotic drug caspase inhibitor) z-DEVD-fmk is a substrate of Pgp/MRP1 and is pumped out from MDR cells [7]. The combination of flavopiridol and z-DEVD-fmk kills MDR cells, while sparing parental cells (Figure 2).

Flavopiridol can be substituted by other apoptosis-inducing drugs that also are NOT substrates of Pgp/MRP [7]. For example, docetaxel (Taxotere) is NOT an MRP1 substrate and it induces apoptosis in both parental and MRP-expressing cells [8]. Ixabepilone, a clinically available analog of epothilones, is NOT a substrate of PgP [9, 10]. The mitosis-specific drugs Taxotere (and other taxanes) and Ixabepilone kill preferentially proliferating cells, by causing mitotic arrest, when the cells enter mitosis. At low (cytostatic) concentrations, DNA-damaging drugs (doxorubicin, actinomycin D and etoposide) arrest HL60 cells in G2 phase of the cell cycle [6, 7]. By arresting parental HL60 cells prior to mitosis, these drugs protect arrested cells from apoptosis caused by mitosis-specific drugs [6]. A combination of Low-DOX and epothilones kills multi-drug-resistant cells, while sparing parental cells [6].

Combining cytostatic and cell-cycle-specific cytotoxic drugs is called cyclotherapy. For cyclotherapy, resistant cancer cells must be resistant to cytostatic drugs and then they can be killed by a cell-cycle-specific cytotoxic drug. Generally, resistance to cell-cycle-arrest is due to universal dysregulation of the cancer cell cycle due to loss of p53, Rb and other tumor suppressors and overactivation of mitogenic kinases.

### P53-dependent cyclotherapy

The term cyclotherapy was introduced in 2002 [11] to describe a strategy to selectively protect normal cells from cell-cycle-dependent chemotherapy by inducing protective cell-cycle arrest. By 2002, several studies had already demonstrated the feasibility of this approach in cell culture [6, 12–15].

Mutations in p53, the most common alteration in human cancer, renders cancer cells resistant to cell-cycle arrest by p53-inducing drugs. For example, low concentrations of doxorubicin induce p53 and cause cell cycle arrest in parental HCT116 cells but not in HCT116-p53−/− cells lacking p53 [13]. Then, treatment with paclitaxel killed HCT116-p53−/−, whereas parental cells were protected by cell-cycle arrest [13].

**Figure 3:**
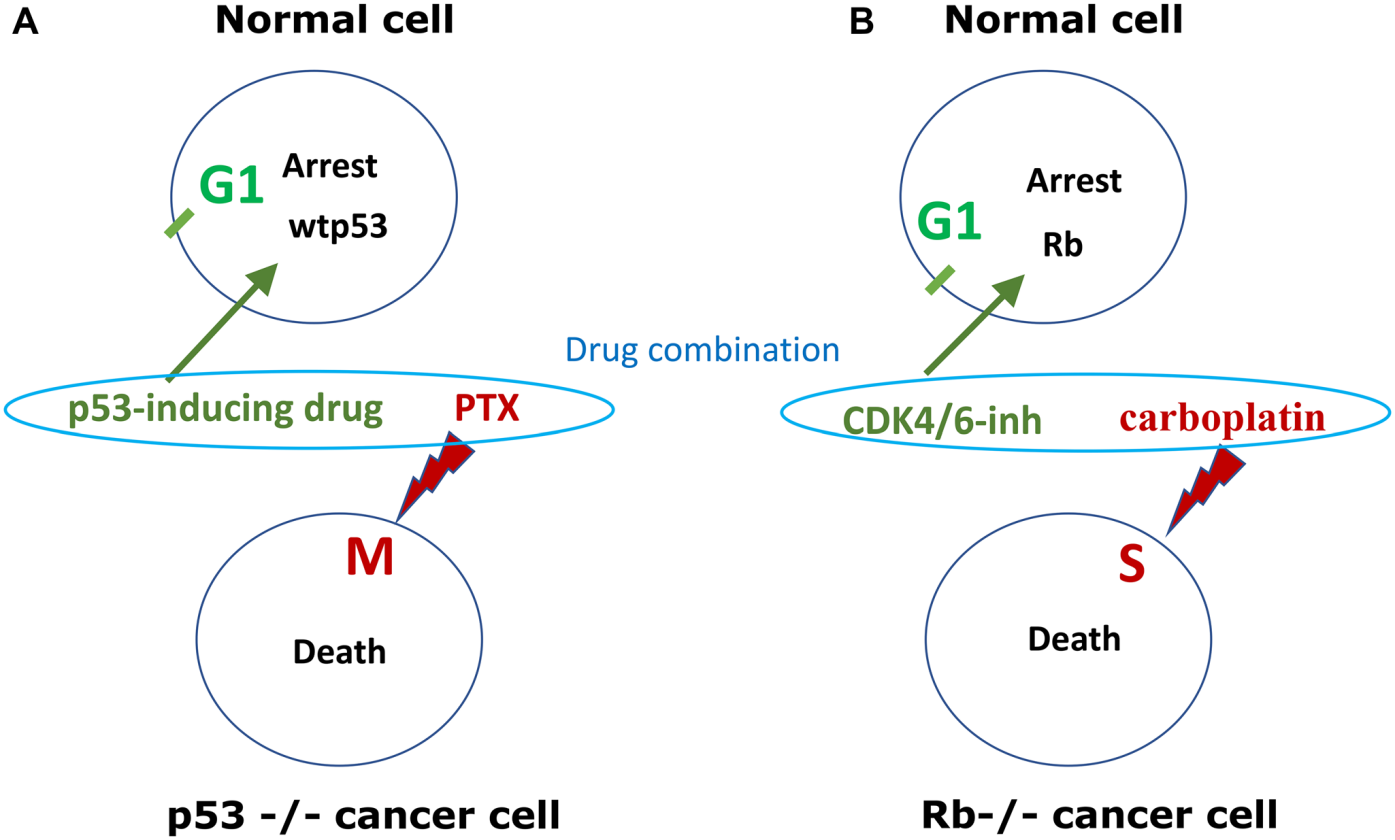
p53-dependent and Rb-dependent cyclotherapy. (**A**) p53-dependent cyclotherapy. Normal cell and cancer cells, lacking wild type p53 (p53−/−), are treated by low dose p53-inducing drugs (e.g., nutlin-3, DOX, ActD) and then treated byTaxol (PTX), which kills cells in mitosis. Protective drugs (green), lethal drugs (red). Induction of p53 causes G1 arrest and protects cells from mitosis-specific lethality of PTX. (**B**) Rb-dependent cyclotherapy. Cancer cells lack. Normal cells and cancer cells, lacking Rb (Rb−/−), are treated by a combination of low dose CDK4/6 inhibitor (e.g., trilaciclib, palbociclib) and DNA-damaging chemotherapy (5-FU, carboplatin, etoposide), which kill cells in S-phase. Protective drug (green), lethal drug (red). CDK4/6 inhibitor causes G1-arrest in normal cells, protecting cells from S-specific lethality of chemotherapy.

In these studies, “parental cells” represent normal cell-cycles because normal cells always have wt p53. P53-dependent cyclotherapy can be used for the protection of normal cells, when tumor cells lack p53 (Figure 3A).

Low concentrations of DNA-damaging drugs such as doxorubicin, etoposide and actinomycin D induce p53-dependent G1 and G2 arrest in normal and cancer cells with wild type p53, protecting them from cell death caused by S-phase or M-phase specific chemotherapy [13, 16, 17]. Inducers of p53 protected cells with wtp53 from both S-phase specific and M-specific chemotherapy, without protecting p53-deficient cancer cells [18–20]. However, even at low concentrations, DNA-damaging drugs can cause p53-independent arrest in some cell lines. For example, HL60 leukemia cell line lacking p53 is still arrested in G2 phase by doxorubicin via Chk1-dependent checkpoint [21]. UCN-01, an inhibitor of multiple kinases including Chk1, overrode DOX-induced G2 arrest, thus propelling these p53-deficient cells from G2 to mitosis. Once they entered mitosis, cells were killed by PTX [21].

The p53-dependent cyclotherapy was developed using different inducers of p53 that protected normal cells from S-phase and mitosis-specific chemotherapeutics [18–31]. Unfortunately, p53-inducing DNA-damaging protective drugs have a narrow protective window. For example, doxorubicin is protective at 20–100 ng/ml and becomes cytotoxic above 100 ng/ml in cell culture [32].

In contrast, inhibitors of Mdm-2, such as nutlin-3a, are not genotoxic. Activation of p53 by Nutlin-3 leads to G1 and G2 arrest and protects proliferating normal cells from mitotic inhibitors such as paclitaxel [33]. Nutlin-3 did not protect cancer cells with mutant p53 from paclitaxel-induced apoptosis [33]. Selective protection of normal cells with Nutlin-3 from cytotoxicity of mitotic inhibitors such as paclitaxel and nocodazole, as well as PLK1 and aurora kinase inhibitors, has been confirmed and further extended in numerous studies [16, 19, 34–36].

Importantly, nutlin-3 can protect normal bone marrow cells *in vivo*, without protecting cancer cells, in mice treated with mitosis-specific chemotherapy [34]. Thus, mice treated with BI-2536 (PLK1 inhibitor) developed neutropenia. Oral administration of Nutlin-3 efficiently protected the mice from this neutropenia [34].

Recombinant human IL-1 receptor antagonist (IL-1Ra) causes protective arrest of hematopoietic cells through a p53-dependent cyclotherapy mechanism [22, 29, 37]. IL-1Ra reduces lethality and bone marrow toxicity of 5-fluouracil in mice [37] and selectively protects intestinal crypt epithelial cells from chemotoxicity, but not tumor cells [22]. IL-1Ra reduces thymus toxicity of 5-azacytidine in mice [38].

### P53-independent cyclotherapy

As an example of p53-independent cyclotherapy, kinase inhibitor staurosporine [15] and its analog UCN-01, kinase selectively protects normal cells in Rb-dependent manner [39]. Normal mammary epithelial cells and breast cancer cells were treated with low (cytostatic) concentrations of staurosporine, arresting normal cells in G1 without affecting cancer cells. This arrest protected normal cells from doxorubicin and camptothecin [15]. UCN-01 reversibly arrested normal gut epithelial cells, protecting them from cytotoxicity of 5-FU, decreased side effects and enhanced therapeutic efficacy, decreased tumor size and increased survival [40].

Low doses of AG1478, an inhibitor of EGF receptor kinases, arrested proliferation of immortalized breast cells but not EGF-independent cancer cells. Pretreatment with AG1478 selectively protected non-cancerous cells from paclitaxel [14].

Flavopiridol, a pan-CDK inhibitor, is highly cytotoxic at high concentrations. At low concentrations, flavopiridol protects p21-sensitive cells from paclitaxel [41].

### Cyclotherapy with CDK4/6 inhibitors

Selective protection of normal cells with CDK4/6 inhibitors is a clear-cut example of cyclotherapy (Figure 3B). In 2010, Johnson et al. showed that treatment of mice with PD0332991 (palbociclib), a CDK4/6 inhibitor, caused reversible quiescence of early hematopoietic stem/progenitor cells (HSPCs) but not most other cycling cells in the bone marrow or other tissues [42]. Pharmacological quiescence decreased the hematopoietic toxicity of total body irradiation [42, 43]. Palbociclib (PD0332991) also protected bone marrow from carboplatin, improving blood cell counts in carboplatin treated mice. As expected, it decreased antitumor activity of carboplatin against *Rb*-competent tumors but did not protect *Rb*-deficient tumors, which were resistant to palbociclib [1]. Thus, palbociclib protected bone marrow without protecting Rb-negative tumors. The authors concluded that “CDK4/6 inhibitors should not be combined with DNA-damaging therapies, such as carboplatin, to treat tumors that require CDK4/6 activity for proliferation” [1]. It was also shown that palbociclib prevented radiation-induced lethal intestinal injury in mice [44].

In 2015, palbociclib was approved by the FDA, not for protection of normal cells but for the treatment of estrogen receptor (ER)-positive, HER2-negative advanced breast cancer as initial endocrine-based therapy in postmenopausal women.

To protect normal cells, the new CDK4/6 inhibitor G1T28 (trilaciclib) has been developed [2, 45]. Like palbociclib, trilaciclib reversibly decreases proliferation of bone marrow hematopoietic stem/progenitor cells. Trilaciclib does not decrease the efficacy of cytotoxic chemotherapy on Rb1-deficient tumors, whose proliferation is CDK4/6-independent [2, 45].

In a randomized, double-blind, placebo-controlled Phase II study in patients with small cell lung cancer treated with carboplatin, etoposide and atezolizumab (E/P/A), trilaciclib decreased the occurrence and duration of severe neutropenia and improved red blood cell counts, platelet counts and quality of life. Trilaciclib did not affect antitumor activity of chemotherapy [46]. Other clinical trials also demonstrated myeloprotection [47–50]. Trilaciclib reduced topotecan-induced myelosuppression and improved safety profile and quality of life without detrimental effects on antitumor efficacy [48]. Trilaciclib decreased the need in supportive care interventions for chemotherapy-induced myelosuppression in patients with small cell lung cancer [51].

In February 2021, trilaciclib was approved by the FDA to decrease the incidence of chemotherapy-induced myelosuppression in adult patients when administered prior to a platinum/etoposide-containing regimen or topotecan-containing regimen for extensive-stage small cell lung cancer (ES-SCLC). Clinical studies in breast cancer, colorectal cancer and small cell lung cancer are underway in several countries [52].

### CDK4/6 inhibitors for myeloprotection in cancers treatable with CDK4/6 inhibitors (proposal)

The use of trilaciclib for myeloprotection is a success story for the cyclotherapy concept. There are some limitations. While palbociclib (and several other CDK4/6 inhibitors) is approved for cancer therapy but not for myeloprotection, trilaciclib is approved for myeloprotection but not for cancer therapy. This may unnecessarily complicate the use of CDK4/6 inhibitors as protectors. The use of the same drug for two purposes may have several advantages. First, we would know the difference between therapeutic (anti-cancer) and myeloprotective doses. In theory, myeloprotective doses must be lower than anti-cancer doses. Anti-cancer doses cause myelosuppression, whereas protective doses cause reversible G1 arrest. Second, if a drug is already used for the treatment, it may not require government (FDA) approval to be used in the same patient at lower doses (for myeloprotection). This extends its use from lung cancer to other cancers. (Later, we will specifically discuss breast cancer).

As an additional advantage, oral administration palbociclib is more convenient than intravenous administration of trilaciclib.

### How to introduce palbociclib for myeloprotection and ensure that it does not protect tumors (proposal)

**Figure 4:**
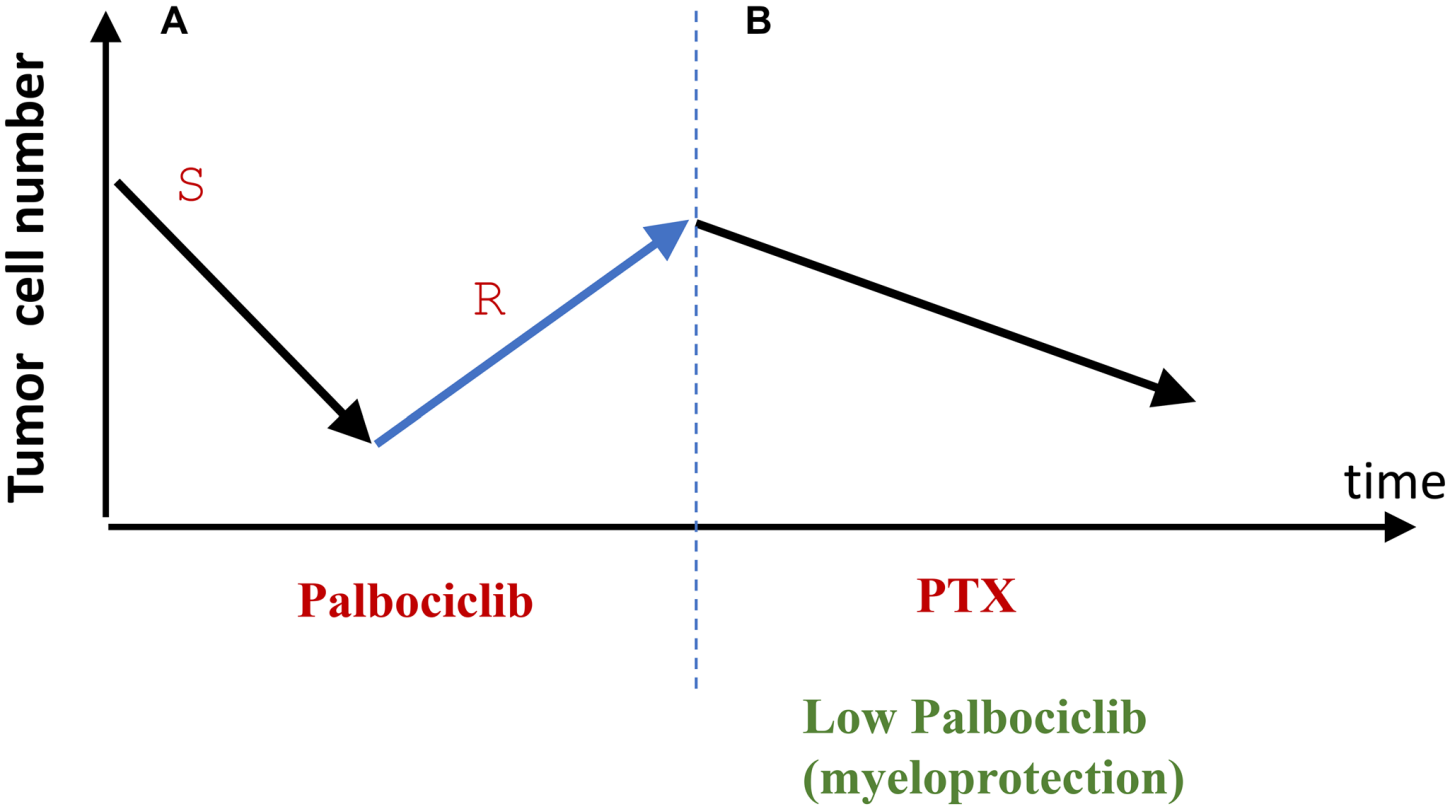
From tumor relapse to selective protection of normal cells (proposal). (**A**) At therapeutic doses, palbociclib causes therapeutic response by eliminating palbociclib-sensitive (S) cancer cells. Selection for resistant cancer cells (R) leads to relapse and tumor progression. (**B**) Relapsed palbociclib-resistance is treated with Taxol (PTX). Palbociclib is used to protect normal cells from PTX. The cancer cells will not be protected because they are palbociclib-resistant.

CDK4/6 inhibitors are usually initially effective for HR+/HER2- metastatic breast cancer. However, resistance is developed. This “therapeutic failure” is an opportunity (Figure 4). If a tumor grows at anti-cancer doses of palbociclib, it means palbociclib does not cause cell cycle arrest. Even more certainly, a lower dose would not cause arrest. This ensures protection of normal cells selectively, without affecting cancer cells. (Note: normal cells do not develop resistance).

In contrast, the current way of use for trilaciclib in lung cancer is partly a lottery. It is assumed that, in each and every patient, cancer is trilaciclib-resistant and will not be protected from chemotherapy. Trilaciclib does not decrease the efficiency of chemotherapy. Anti-cancer efficiency of therapy was the same in groups of patients with and without trilaciclib [46]. However, it is possible that trilaciclib potentiates therapy in some patients and antagonizes it in others. (No decrease in efficacy can be then detected in clinical trials). The latter sub-group should not be treated with trilaciclib. Unfortunately, exactly who belongs in this sub-group cannot be known.

This undesirable scenario can be avoided in a “role reversal” proposal discussed in this section. In patients who failed therapy with palbociclib (used as anti-cancer drugs), lower (protective) doses of palbociclib cannot cause protective arrest in palbociclib-resistant cancer.

### Prevention of cell senescence

The protective arrest must be reversible (quiescence). Palbociclib induces reversible arrest in bone marrow *in vivo* [42]. In cells with overactivated mTOR, palbociclib (PD0332991) induces irreversible senescence [53, 54], because mTOR drives geroconversion from quiescence to senescence. By inhibiting mTOR, rapamycin and everolimus ensure quiescence in palbociclib-treated cells [53]. Similarly, nutlin-3a (Mdm-2-Inhibitor) causes quiescence and senescence depending on the mTOR activity [55]. A combination of mTOR inhibitors with nutlin-3a [56] and CDK4/6 inhibitors [53] may be considered for quiescence in normal cells.

### Proposal: a combination of mTOR and CDK4/6 inhibitors to mitigate side effects of chemotherapy

Everolimus, a rapamycin analog, is approved for treatment of advanced HR+, HER2- breast cancer. This is exactly the same type of cancer that is also treated with palbociclib. In some studies, therapies with everolimus and palbociclib were used in sequence: when one of them failed, the other one was used in sequences [57–59]. In one trial, palbociclib was given first, followed by everolimus [57]. In another study, the sequence was the opposite: everolimus was followed by palbociclib [58].

Proposal: A combination of low doses of both everolimus plus palbociclib for selective protection of normal cells against chemotherapy with taxanes in patients who failed everolimus/palbociclib as a prior therapy. Palbociclib-resistant cells are sensitive to taxanes (paclitaxel and docetaxel) [60]. Taxanes are widely used for the treatment of breast cancer [61, 62]. Notably, CDK4/6 inhibition mitigates stem cell damage in an *in vitro* model for taxane-induced alopecia [63]. Prevention of hair loss is easily observable by the patient.

### Failed therapy, repurposing, selection for drug sensitivity

If a targeted drug fails (and a resistant tumor grows despite the treatment), then the failed drug can be considered (at lower doses) for selective protection of normal cells from cell-cycle-dependent chemotherapy.

Probably, side effects of targeted therapy, when it is used to treat cancer, may predict which normal cells will be protected, when it is used as a protector. For example, more myelotoxicity is observed with palbociclib, but more gastrointestinal toxicity is observed with abemaciclib [64]. As a protector, palbociclib mainly protects bone marrow cells [1]. In theory, abemaciclib may protect epithelial cells from cell-cycle-specific chemotherapy. Similar, EGF-R inhibitors display skin, hair and gastrointestinal toxicity, and may be predicted (at lower doses) to mitigate these side effects caused by chemotherapy.

For example, CDK4/6 inhibitors cause myelosuppression in anti-cancer doses but prevent chemotherapy-induced myelosuppression in protective doses.

Targeted therapeutics that failed clinical trials can be repurposed for protection of normal cells [65]. For example, UCN-01 failed clinical trials as monotherapy [66]. UCN-01 showed no efficacy in combinations with DNA-damaging chemotherapy in many clinical trials [67]. In mice, UCN-01 protected gut epithelial cells from DNA-damaging drug 5-FU [40].

Mdm-2 inhibitors unsuccessfully struggle to be approved as an anti-cancer therapy for almost two decades [68, 69]. Besides, Mdm-2 inhibitors inevitably select for mt p53 by suppressing growth of cancer cells with wt p53 [70, 71]. As it was asked, “can we overcome resistance to mdm-2 inhibitors?” [69]. Maybe yes or maybe not. But why should we make the task of cancer therapy so difficult? It’s already difficult to treat cancer even without resistance to therapy. When resistance develops, why not to switch to a different kind of therapy (and simultaneously exploit resistance to mdm-2 inhibitors for selective protection of normal cells from this therapy)?

**Figure 5:**
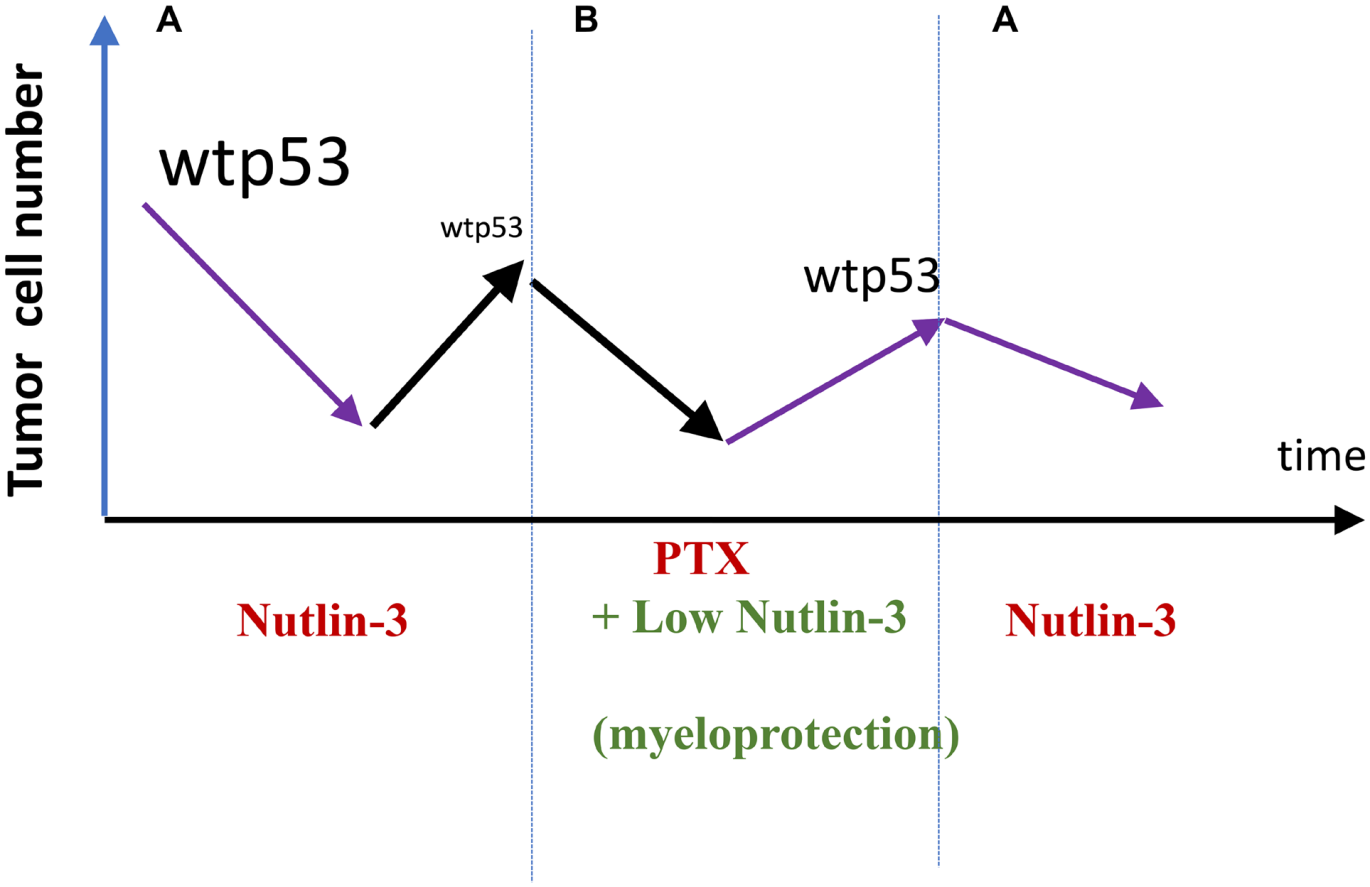
Exploiting selection for resistance for (and against) nutlin-3 (proposal). (**A**) Nultin-3 causes response in wt p53-expressing tumors. By killing cells with wt p53, it selects for loss of wt p53 (nutlin-3-resistance). (**B**) Relapsed nutlin-3-resistance tumors is treated with Taxol. Low doses of nutlin-3 are used to protect normal cells from Taxol (PTX). The cancer cells will not be protected because they are nutlin-3-resistant. A combination nutlin-3 plus PTX may selected for clones with wt p53. Then (A) repeat.

Consider a scenario. By inducing wt p53, an mdm-2 inhibitor (for example, nutlin-3) can kill or arrest cancer cells with wt p53. This must select for resistant cells, harboring mutant p53, as an example [72, 73]. Then, lower doses of nutlin-3 can be used to selectively protect normal cells (for example, bone marrow cells) from chemotherapy [34]. This combination may selectively kill cancer cells resistant to nutlin-3, selecting for cancer cells sensitive to nutlin-3 (Figure 5). When therapy with the combo fails, the tumor is nutlin-3-sensitive again and the cycle can be repeated (Figure 5).

### Therapeutic engineering

We may design therapeutic multi-drug combinations with effects distinct from the effects of each drug alone [56]. For example, in fibrosarcoma cell line with IPTG-inducible p21, (a) IPTG causes irreversible senescence, (b) mitosis-specific drugs (nocodazole, paclitaxel) cause cell death in mitosis and (c) rapamycin inhibits cell growth [56]. A combination of these three drugs, in the right sequence, cancel some effects of each other and the cells emerge healthy and proliferating (when the drugs are washed out).

Added together with IPTG, rapamycin prevents senescence, preserving reversible quiescence instead. This pharmacological quiescence prevents mitotic arrest and cell death otherwise caused by Nocodazole. When all three drugs are removed, the cells restart proliferation. This amazing outcome depends on the exact drug sequence. For example, addition of IPTG after nocodozole (not before) does not prevent cell death [56].

In therapeutic engineering, the same drug can play roles of either cytotoxic or protective drugs depending on doses, sequences and cancer cell genetic profile. For example, by inducing G1-cell-cycle arrest, UCN-01 plays a protective role in normal cells against S-phase-specific chemotherapy [40]. On the other hand, by abrogating p53-independent G2-checkpoint, UCN-01 prevents protection of cancer cells with mutant-p53 from mitosis-specific combination [21]. This combination includes three drugs: low concentrations of DNA-damaging drugs (doxorubicin, etoposide), followed by UCN-01 to propel cells from G2 to mitosis and then, exposing them to paclitaxel [21].

Different protectors (and their combinations) will be needed to protect different kinds of normal cells, in different cancers and different types of cytotoxic therapy. For example, CDK4/6 inhibitors were initially envisioned to (a) protect bone marrow cells (not necessarily all types of normal cells) (b) from chemotherapy with S-phase-specific drugs such as 5-fluorouracil and carboplatin (c) in patients with Rb1-negative cancers (resistant to CDK4/6 inhibitors) [1, 2, 45].

### Synergistic/antagonistic combinations

**Figure 6:**
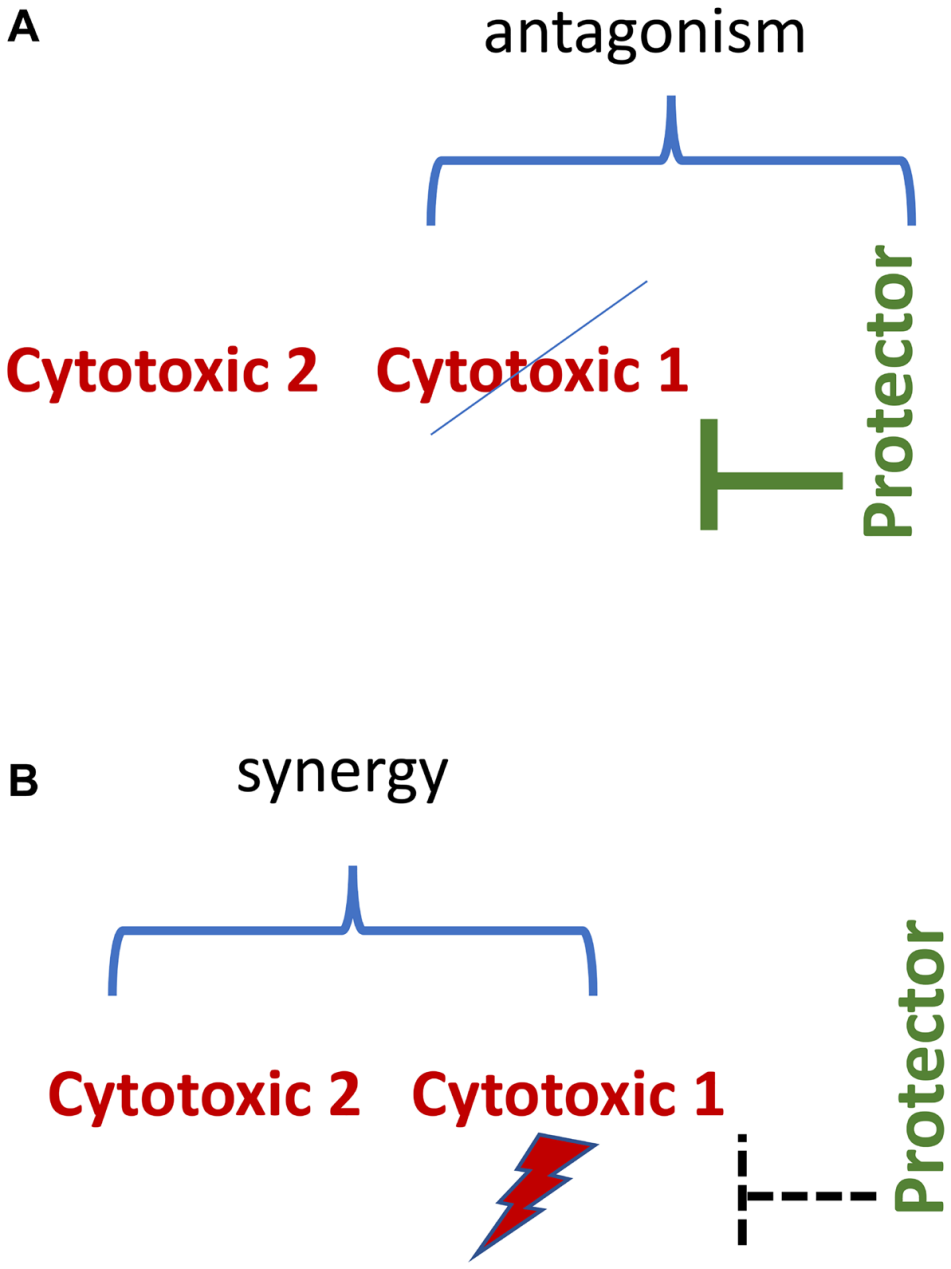
Synergistic/antagonistic combinations. (**A**) Normal cell. Protecting drug antagonizes cytotoxic 1 drug. (**B**) Protector-resistant cancer cell. Cytotoxic drugs 1 and 2 drugs are synergistic.

When normal cells are protected, we can add an enhancing drug that potentiates cytotoxic therapy against cancer cells [74, 75]. This may improve the efficacy of cancer therapy (Figure 6).

For the last 60 years, thousands of synergistic chemotherapeutic drug combinations have been described. Medline search on key words “synergistic+chemotherapy+cancer” retrieved 25,000 publications.

https://pubmed.ncbi.nlm.nih.gov/?term=synergistic+chemotherapy+cancer

This is just a fraction of all publications on the topic. However, synergistic combinations can be synergistically toxic to the patient [76].

In theory, selective protection of normal cells from one of the two synergistic drugs may be sufficient in protection from the synergistic combination. For example, G1-arrest protects against cytotoxicity of mitotic inhibitors such as paclitaxel and therefore against paclitaxel-based synergistic combinations. Such three-drug combinations are antagonistic-synergistic (Figure 6).

It is also possible to design two-drug antagonistic-synergistic combinations.

In such combinations, a targeted drug antagonizes the cytotoxic drug in normal cells and potentiates it in cancer cells. For example, geldanamycin (GA), a drug which destabilizes Hsp90-associated proteins, depletes cells of Bcr-Abl and some other oncogenic kinases. These kinases render cancer cells resistant to chemotherapeutics such as paclitaxel and doxorubicin. GA sensitized Bcr-Abl-expressing cells to doxorubicin and paclitaxel. In contrast, parental cells lacking oncogenic Bcr-Abl were sensitive to chemotherapeutics. GA rendered these cells resistant to chemotherapeutics by inducing hsp-70, an anti-apoptotic protein [77, 78].

### Restrictive targeting of specific clone

Given tumor heterogeneity, drug combinations should be aimed at the deadliest cancer clone. For example, in a tumor with wt p53 and mutant p53 clones, a combination of nutlin-3 and paclitaxel (PTX) is aimed at mutant p53 clones, in order to spare normal cells (all normal cells have wt p53). One of the characteristics of the deadliest clone is proliferation, because, if cancer cells cannot proliferate, they are not immediately dangerous [79]. In theory, it is sufficient to target cycling cells to control cancer.

### Protection beyond cyclotherapy: Inhibitors of apoptosis

Pifithrin-alpha, inhibitor of wt p53, protects mice from side effects of genotoxic chemotherapy [80, 81].

The most important group is inhibitors of caspases. Unfortunately, none of caspase inhibitors or other inhibitors of cell death are clinically approved. Caspase inhibitors struggle to be approved for treatment of non-oncologic diseases and have failed so far. For example, Emricasan (IDN-6556, PF-03491390) is a caspase inhibitor invented in 1998 by Idun Pharmaceuticals. It has been granted fast track designation by the FDA for the treatment of non-alcoholic steatohepatitis cirrhosis [82, 83]. In two clinical trials, emricasan did not demonstrate a beneficial effect of non-alcoholic steatohepatitis-related cirrhosis and fibrosis [82, 83]. In contrast, we need caspase inhibitors for selective protection of normal cells from apoptosis-inducing chemotherapy, without protecting MDR cancer cells, for example [7].

### Brain tumors (proposal)

As we discussed, a combination of a cytotoxic chemotherapy that is not a substrate of PgP and a protective drug that is a substrate of PgP can selectively kill Pgp-expressing cells, while other cells are protected [7]. A similar approach can be suggested to eliminate systemic side effects of chemotherapy in patients with brain tumors. In simple terms, brain tumors are separated from the rest of the body by blood brain tumor barrier (BBB), which involves ABC transporters such as Pgp, MRP and BCRP [84, 85]. An antagonistic drug combination can be used to target brain tumors, while sparing cells elsewhere. For example, temozolomide, an apoptosis-inducing drug [86], crosses the blood-brain barrier and is widely used for treatment of brain tumors [87]. By killing proliferating normal cells, temozolomide causes systemic side effects such as myelosuppression, hair loss and mucositis. Caspase inhibitors that cannot cross the blood-brain barrier can be used to protect normal cells from temozolomide-induced side effects. Brain-impermeable caspase inhibitor plus temozolomide.

More generally, antagonistic combinations include a brain-permeable cytotoxic drug and the brain-impermeable antagonist. This antagonistic drug combination is expected to mitigate the systemic effects of chemotherapy but retains the efficacy of chemotherapy in brain cancer.

Any protectors that do not cross the blood brain tumor barrier can be used to mitigate systemic side effects of chemotherapy. In the case of temozomide, the use of a brain-impermeable inhibitor of alkylation would be the best option.

Systemic side effects of temozomide (and other chemotherapy for brain tumors) may be mitigated based on the cyclotherapy approach. CDK4/6 inhibitors such as palbociclib do not cross the blood brain barrier [88–90]. Palbociclib and trilaciclib, CDK4/6 inhibitors, cause G1 cell-cycle arrest in bone marrow cells, thus protecting them from chemotherapy. Given that they are already clinically available, it will be easy to design a study to evaluate side effects caused by temozomide, given with or without CDK4/6 inhibitors to the same patient.

Furthermore, protective combinations may include inhibitors of CDK4/6, mTOR, mdm-2, and caspase, all together. Such protective cocktails may eliminate most systemic side effects: from myelosuppression to hair loss.

What would happen when resistance to telosmoside (or other chemotherapy) develops? Enormous research efforts have been focused on overcoming such a resistance and this is not the topic of this article. Alternatively, resistance can be exploited. Also, in theory, resistance may not develop at all, because temozomide may be used at higher doses and, most importantly, in a synergistic-antagonistic combination to start with. However, until protection of normal cells is implemented, a detailed discussion of further strategies is too preliminary.

## CONCLUSION AND FURTHER SUGGESTIONS

Selective protection of normal cells may transform therapy of cancer. Especially when targeted therapy fails, patients can be treated with therapy that exploits this resistance. Some targeted drugs, at low doses, can be repurposed as protectors. When normal cells are protected, more potent synergistic drug combinations could be designed, and potentially higher doses or treatment duration could be used. Standard therapies that select for drug resistance can be applied in sequence. Repeating such cycles may extend the life of a cancer patient (see Figure 5).

Potential opportunities are enormous, but they cannot be implemented in one step. The first step should be mitigating chemotherapy side effects in a variety of cancers. This article focuses on a few examples that may be implemented now. The approval of the CDK4/6 inhibitor trilaciclib for myeloprotection in patients with lung cancer shows that it is possible. One approach is to repurpose targeted drugs such as CDK4/6 inhibitors (palbociclib, abemaciclib) and everolimus/sirolimus, when therapy with these drugs fail. This will ensure that the tumor will not be protected because the resistance is proven by therapeutic failure in the same patient. Another approach is using caspase inhibitors and Mdm-2 inhibitors for selective protection of normal cells, based on the lessons of clinical development of trilaciclib. At low doses, Mdm-2 and caspase inhibitors in combination with everolimus may be especially useful to mitigate side effects of mitosis-specific chemotherapy, such as Vinca drugs and Taxanes, in patients with mutant p53 tumors. As a special approach, any brain-impermeable protective drugs, including trilaciclib, can in theory be used to mitigate systemic side effects of brain-permeable cytotoxic drugs such as temozolomide in the treatment of brain tumors. These are most obvious examples of immediate clinical implementation of the concept of “exploiting drug resistance.” A variety of other clinical implementations can be suggested by readers of this article.
